# 213. Assessment of Compliance with Order Set and Bundle for Management of *Staphylococcus aureus* Bacteremia

**DOI:** 10.1093/ofid/ofab466.415

**Published:** 2021-12-04

**Authors:** Ryan R Flynn, Marilee Obritsch, Veronica Lesselyoung, Joe Strain, John Kappes

**Affiliations:** Monument Health, Hartley, Iowa

## Abstract

**Background:**

*Staphylococcus aureus* (*S. aureus*) is an aerobic gram-positive coccus that causes a variety of infections. *S. aureus* bloodstream infections, also known as bacteremias, have significant morbidity and mortality and are difficult to eradicate. A single-center study showed a 9.4% recurrence rate for *S. aureus* bacteremia, despite adequate treatment. The Infectious Disease Society of America (IDSA) recognizes the seriousness of *S. aureus* infections, particularly methicillin-resistant *S. aureus* (MRSA), and has released guidance for treatment of these infections. Guidance for *S. aureus* bacteremias include identification and removal of the source and early optimization of antibiotics. Serial imaging and laboratory monitoring, including repeat blood cultures, are also necessary to establish the duration of therapy, ensure microbiologic eradication, and reduce the risk of long-term complications. Due to the complexity of *S. aureus* bacteremia, early involvement of infectious diseases (ID) specialists is strongly recommended.

**Methods:**

This retrospective, single-center study was designed to evaluate the current management of *S. aureus* bacteremias, including compliance to the elements of the *S. aureus* order set and bundle. Patients 18 years and older who had a positive blood culture for *S. aureus* were included in this study. Recurrence of *S. aureus* infection was assessed at 6 months. Data was analyzed to compare patients with and without ID consults.

**Results:**

Eighty-four patients met inclusion criteria. ID consultation resulted in a higher percentage of patients achieving 100% compliance with the bundle elements compared to patients without ID consults (73% vs 25%, respectively; p=0.009). For further breakdown of compliance see Table 1. No statistical difference was detected in recurrence rates (11% vs 33%, respectively; p=0.18) or mortality (8% vs 25%, respectively; p= 0.17) possibly due to the small sample size.

Table 1. Outcomes

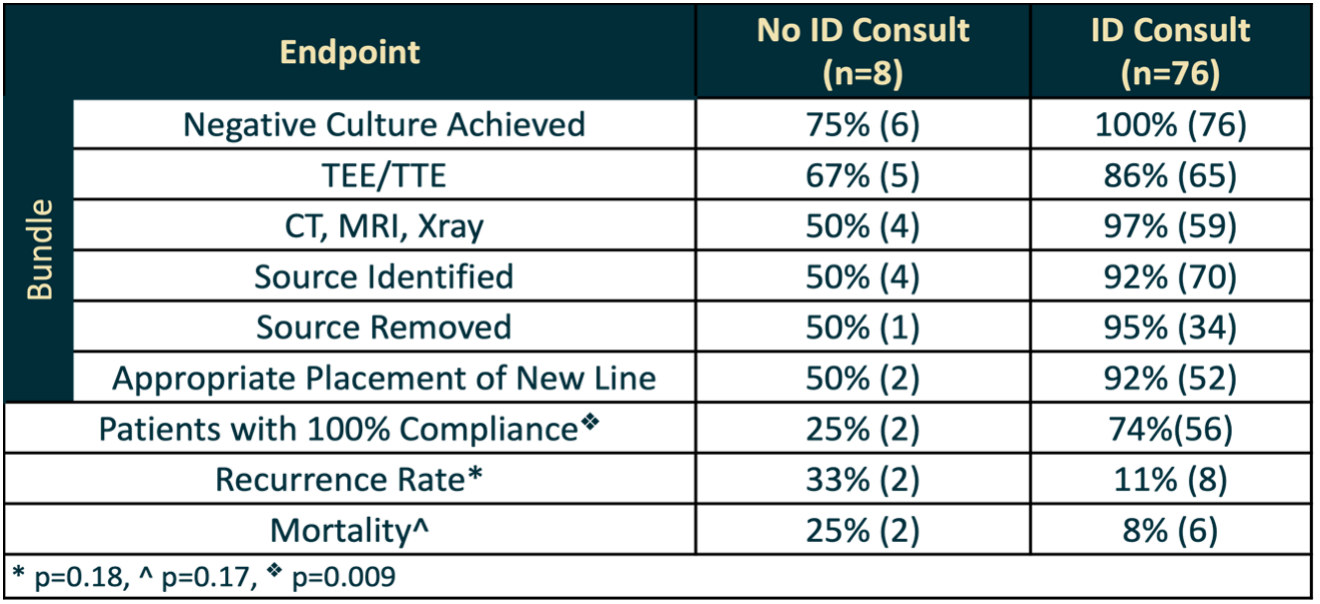

**Conclusion:**

ID specialist involvement for the treatment of *S. aureus* bacteremia resulted in greater compliance with the *S. aureus* bacteremia bundle. No statistical difference in recurrence or mortality rates was detected.

**Disclosures:**

**All Authors**: No reported disclosures

